# Neural correlates of co-occurring pain and depression: an activation-likelihood estimation (ALE) meta-analysis and systematic review

**DOI:** 10.1038/s41398-022-01949-3

**Published:** 2022-05-11

**Authors:** Carmen Jiamin Zheng, Sarah Van Drunen, Natalia Egorova-Brumley

**Affiliations:** 1 The University of Melbourne, Parkville, VIC Australia; 2 The Florey Institute of Neuroscience and Mental Health, Parkville, VIC Australia

**Keywords:** Diagnostic markers, Human behaviour

## Abstract

The relationship between pain and depression is thought to be bidirectional and the underlying neurobiology ‘shared’ between the two conditions. However, these claims are often based on qualitative comparisons of brain regions implicated in pain or depression, while focused quantitative studies of the neurobiology of pain-depression comorbidity are lacking. Particularly, the direction of comorbidity, i.e., pain with depression vs. depression with pain, is rarely addressed. In this systematic review (PROSPERO registration CRD42020219876), we aimed to delineate brain correlates associated with primary pain with concomitant depression, primary depression with concurrent pain, and equal pain and depression comorbidity, using activation likelihood estimation (ALE) meta-analysis. Neuroimaging studies published in English until the 28th of September 2021 were evaluated using PRISMA guidelines. A total of 70 studies were included, of which 26 reported stereotactic coordinates and were analysed with ALE. All studies were assessed for quality by two authors, using the National Institute of Health Quality Assessment Tool. Our results revealed paucity of studies that directly investigated the neurobiology of pain-depression comorbidity. The ALE analysis indicated that pain with concomitant depression was associated with the right amygdala, while depression with concomitant pain was related primarily to the left dorsolateral prefrontal cortex (DLPFC). We provide evidence that pain and depression have a cumulative negative effect on a specific set of brain regions, distinct for primary diagnosis of depression vs. pain.

## Introduction

Pain-depression comorbidity is associated with worse clinical outcomes and high treatment costs [1]. Up to 65% of patients with depression experience pain [2], and up to 61% of chronic pain patients suffer from depression [3]. Although highly prevalent, the underlying mechanism of pain-depression comorbidity is not well understood. Available pharmacological treatments that target both pain and depression are costly and provide only modest benefits. Participants with comorbid chronic pain and depression specifically report fewer functional benefits from antidepressant use, lower benefits from sertraline, escitalopram and venlafaxine compared to participants without chronic pain, and lower benefits when taking sertraline, escitalopram and citalopram specifically for chronic pain [4]. The use of antidepressants in chronic pain is associated with moderate efficacy, e.g., ‘limited’ effect in fibromyalgia [5], ‘small and not clinically important’ effects for back pain [6], ‘low certainty evidence’ for an effect in osteoarthritis and sciatica [6]. Pain patients often do not respond well to antidepressants, resulting in higher odds of stopping treatment due to side effects [5, 7], although antidepressants are overall tolerable in low doses [8], and have an increased risk of developing depression or addiction when treated with opioid analgesics [9], likely due to the dysregulation of the amygdala circuitry [10]. As such, better understanding of the depression-pain link is needed for a unified approach to treating comorbid pain and depression.

### Pain with depression vs. depression with pain

The link between depression and pain is believed to be bidirectional [2, 3]. Yet, why comorbidity occurs is not clearly understood. The debate of whether depression is an antecedent or a consequence of chronic pain, sparked by the original suggestion in 1982 that chronic pain could be a variant of depression [11], is long-standing. Previous research on chronic pain suggests that depression is likely a consequence [12] evolving in the background of pain-induced disability. As such, depression is usually treated as a separate disorder, albeit requiring combined treatment [13, 14]. On the other hand, with primary depression there is evidence of conversion to chronic pain [15], for example to low back pain. A large longitudinal cohort study has shown that the presence of depressive symptoms predicts future episodes of low back pain, neck-shoulder pain, and musculoskeletal symptoms compared with those patients without depressive symptoms at baseline [16]. Another study showed that low back pain is more than two times as likely to be reported by individuals with depressive symptoms compared with those without, with 16% converting from pain-free depression to low back pain in one year [17]. Therefore, primary depression with pain and primary pain with depression need to be distinguished to better understand how the comorbidity emerges and can be best treated.

### Neuroimaging of pain and depression comorbidity

Pain and depression are said to ‘share neurobiological bases’ [18, 19], but there is little research directly comparing neural correlates of pain and depression or studying their comorbidity. Studies reporting the overlap have emphasised the role of the amygdala, insula, prefrontal cortex, anterior cingulate, thalamus and hippocampus [20] which are linked with the emotional, sensory and cognitive aspects of pain and depression. While research in humans has been dominated by cortical regions implicated in pain and depression, animal studies have identified specific subcortical brain circuits in chronic pain with depression [21]. The most recent and comprehensive study of pain-depression comorbidity in a chronic pain animal model has specifically revealed a neural circuit involving the dorsal raphe nucleus and amygdala [22], while also confirming amygdala connectivity association with pain-depression comorbidity in human studies.

### Study aims

Although there seems to be a matrix of key structures thought to be involved in both pain and depression, findings are hard to compare due to heterogeneity in study methodologies and sample characteristics. Therefore, the aim of this review was to systematically summarise current evidence to identify neural correlates of comorbid pain and depression using ALE analysis. We also aimed to directly compare the results of studies involving participants with the primary diagnosis of pain and concomitant depression versus participants with the primary diagnosis of depression and concomitant pain. In performing our analyses, we considered neuroimaging techniques and clinical characteristics of the study samples, summarising measures used to assess pain and depression and reported severity of pain and depression.

## Materials and methods

### Data source

A comprehensive systematic search was conducted in PubMed, PsychInfo, Web of Science, Medline and Embase databases to identify eligible citations from inception to the date of 15th August 2020. We repeated the search from the 15th of August 2020 to the 28th of September 2021 to identify any additional studies that appeared over the period of data analysis. Across all databases, the combination of all three concepts (i.e., pain, depression and neuroimaging) were used as search terms (see ‘Search terms by database’ in Supplementary Materials). The use of synonyms and keywords varied slightly between databases due to variations in the architecture of their search engines. Where possible, we ensured that each search was maximally inclusive, such as by exploring all major headings, and manually checking to include/exclude each of their respective subheadings.

### Selection criteria

The review protocol is listed in the PROSPERO register under the registration number CRD42020219876. The process of selecting eligible articles was conducted using Covidence software in accordance with the Preferred Reporting Items for Systematic Review and Meta-Analysis (PRISMA) statement. Two authors (CZ and SVD) screened all titles and abstracts, Cohen’s Kappa was measured. Inclusion criteria for primary studies were: i) the full-text was published in English in a peer-reviewed journal; ii) reported original data; iii) included participants with pain reported as either a clinical diagnosis or based on a self-reported test score; iv) included participants with depression reported as a clinical diagnosis or based on a self-reported test score; v) reported results of brain imaging analyses (i.e., MRI, fMRI, PET, SPECT, MRS, DWI).

A primary study was excluded if i) only animal or paediatric data were reported; ii) it did not focus on neuroanatomical structures or functional networks; iii) participants presented with symptoms of comorbid psychiatric disorders beyond depression; iv) participants had a history of neurodegenerative disease or acquired brain injuries; v) electrophysiological studies (e.g., electroencephalography) due to poorer spatial resolution; vi) treatment efficacy studies. Reviews and opinion articles were included only at the initial screening stage for the purpose of extracting additional studies that were not included in systematic search.

The resulting articles were subject to full-text review, during which studies without a statistical analysis that specifically addressed the co-occurrence of pain and depression with neuroimaging were excluded. This means that studies which only assessed neural correlates of pain and did not in some way associate them with depression scores, or vice versa, would not meet the criteria and were thus excluded.

### Quality assessment

Eligible articles were assessed for quality using the National Institutes of Health (NIH) Quality Assessment Tool for Observational Cohort and Cross-Sectional Studies. Two researchers (CZ and SVD) independently evaluated a 50% subset of the original collection of included studies (34 out of 68), selected by taking every second study in alphabetical order of lead author last names. Any conflicts that arose from the review of 50% of the studies were discussed and resolved between CZ and SVD. The researchers agreed on a unified interpretation of each item of the quality assessment tool and uniformly applied the same approach to the assessment of the remaining studies (conducted by CZ). Given that only 7 of 34 studies had minor discrepancies, we estimate that with the adoption of the unified approach the amount of remaining studies that could possibly result in a disagreement does not exceed 5%.

### Data extraction

Data were extracted from each eligible article by CZ. When necessary, authors were contacted by email to obtain further information on reported data. A total of 14 authors were contacted: 7 returned requested data; 3 did not respond with requested clinical data but the studies were included in systematic review with missing information marked as ‘not reported’, 1 provided an unpublished paper (excluded), 3 provided the full text but the study did not meet the inclusion criteria. For all eligible studies, we extracted participants’ characteristics (e.g., average age, type of pain and depression, status of medication use), quantitative measure of pain, group-level average of pain severity, quantitative measure of depression, group-level average of depression severity, and neuroimaging method used.

For the purposes of ALE meta-analysis, three-dimensional coordinates for brain regions in either Talairach or Montreal Neurological Institute (MNI) space were extracted as follows. Studies were categorised by reported analyses. First, we identified studies where the interaction between pain and depression was measured. Then, we grouped the studies where pain was the primary condition, which involved participants with pain who 1) had pain correlated with depression; 2) were compared with healthy controls where depression was used as a covariate and showed a significant effect. We also grouped studies of individuals who presented with depressive symptoms, such that 1) depression was correlated with pain; 2) depressed participants were compared with healthy controls and the pain measure was used as a covariate and showed a significant effect.

### ALE meta-analysis

To perform quantitative meta-analysis, ALE was conducted using GingerALE, Version 3.0.2 (https://www.brainmap.org/ale/). ALE is a voxel-based method that highlights brain regions of significance by pooling coordinates from across neuroimaging experiments. Specifically, activation foci are modelled as three-dimensional Gaussian probability distributions. The width of these probability distributions represents the spatial uncertainty of the foci in consideration of the study sample size and other within-experiment effects. Modelled activation maps were produced by merging probability distributions of all foci from each experiment. The voxel-wise ALE scores – an indication of the degree of concordance in activation between individual studies – were computed by taking the union of these maps. To assess whether the observed convergence of foci between experiments were the result of “true” activation effects or random noise, these ALE scores were compared against a null distribution that assumes a random spatial association between experiments. A cluster-level family-wise error (FWE) corrected *p-*value of 0.05 was used as the threshold of significance (cluster forming *p* < 0.001; 1000 threshold permutations) as per recommendations by Eickhoff and colleagues [23]. All peak voxel coordinates were recorded in MNI space. If the study reported Talairach coordinates, they were converted into MNI space using Talairach to MNI tool implemented within the GingerALE. For illustration, ALE results were mapped on a standardised anatomical MNI-normalised template using the MRIcron software package (https://people.cas.sc.edu/rorden/mricron/install.html).

## Results

### Literature search

**Fig. 1:**
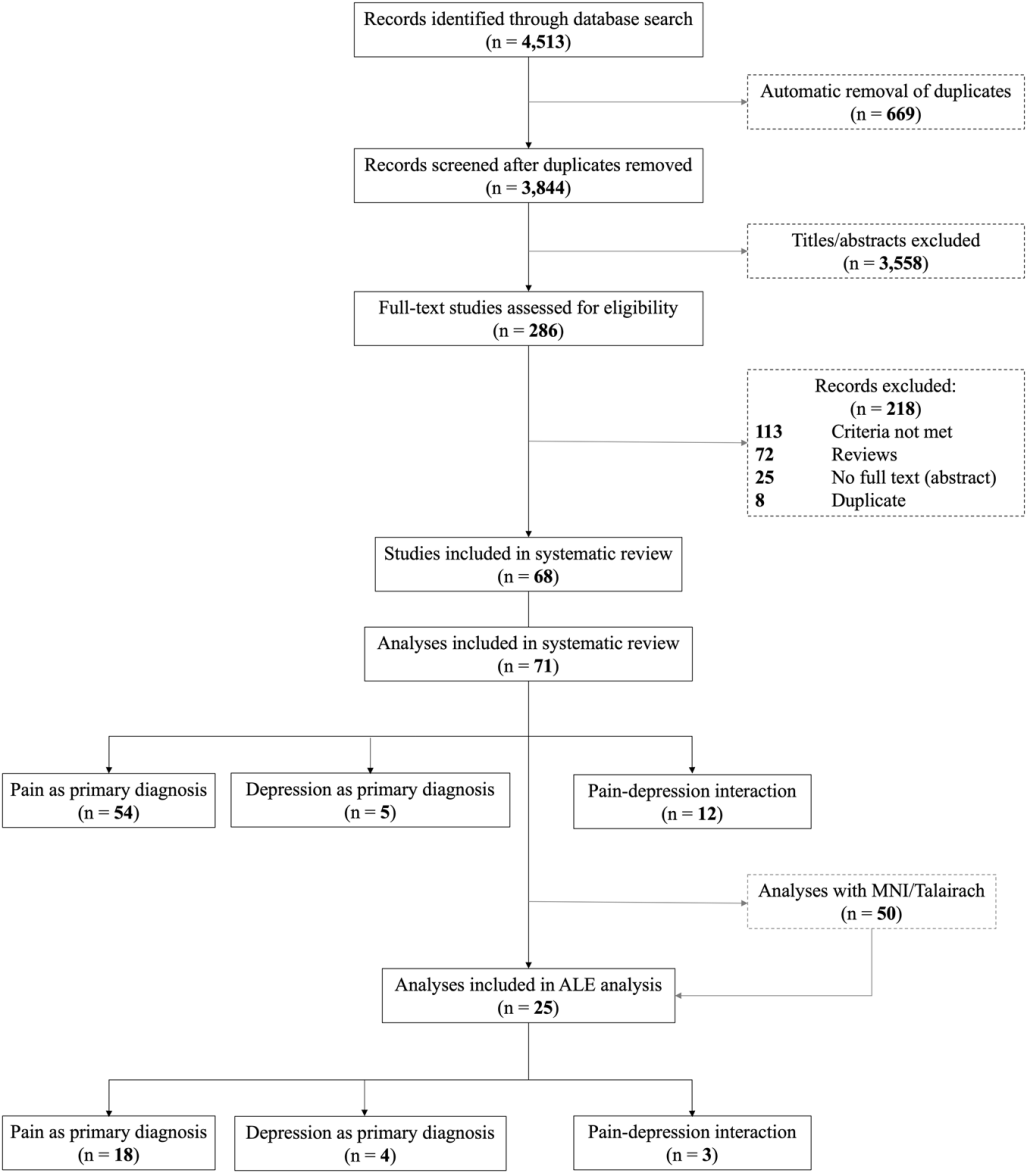
Process of article selection as per PRISMA guidelines. PRISMA flowchart (inception to August 2020 search).

Figure 1 shows the selection process based on PRISMA statement. The initial search identified 4513 citations; no additional articles were retrieved from reference search. After automatic removal of duplicates, 3844 titles and abstracts remained, of which 3558 were excluded; interrater reliability between CZ and SVD was moderate, Cohen’s Kappa = 0.56 [24].

A total of 68 articles met inclusion criteria at full-text assessment and were considered eligible, three of which conducted two separate analyses within the study. Out of the 71 analyses, 12 measured the interaction between pain and depression. Of the remaining analyses, we identified 54 with pain as the primary diagnosis, and 5 with depression as the primary diagnosis. Of the 71 analyses, 50 reported MNI coordinates, 25 reported significant association between pain and depression, which we included in the ALE meta-analysis.

Additional search for the most recent papers (August 2020–September 2021) identified 451 citations, of which 58 duplicates were removed and 362 titles/abstracts were excluded, 31 full texts were assessed but only 2 studies (3 analyses) were eligible, Supplementary Fig. 1. One study (2 analyses) reported MNI coordinates that were used to update the relevant ALE meta-analysis (namely, the Primary Depression contrast).

### Characteristics of studies

Demographic, clinical, and methodological details extracted from the studies are reported in Table 1. Depression severity was determined based on the severity levels established for each depression scale (see ‘Depression scales references’ in Supplementary Materials). In cases where scoring was not known, clinical severity for both pain and depression were approximately categorised based on the reported raw score as proportionate to the maximum score possible, such that minimal (0–25%), mild (25–50%), moderate (50–75%), and severe (75–100%) levels were set. The included studies comprised of 1706 participants considered as having a primary condition of pain (70.9% females), 126 with a primary condition of depression (68.3% females), 402 with both pain and depression (33.4% females), and 1682 healthy controls (68.1% females).

**Table 1:** Details of all 70 (68 + 2 corresponding to 2 search periods) studies included in the systematic review.

Refs.	*N*	Age (year)	Pain type	Pain severity	Dep type	Dep severity	Disease duration (month)	Medication	Comparison type	Direction of effects	Brain region	Neuroimaging	ALE
*Details of 68 studies (inception to August 2020 search) included in the systematic review*													
Adler-Neal 2019 [74]	HC = 76	HC = 27	Exp.	HC = Moderate	Trait	HC = Minimal	NA	Exc	Pain/Dep	Pos Moderation between dep & pain	S2 Operculum Insula OFC VLPFC	task-fMRI	N
Albrecht 2019 [75]	P = 25 HC = 27	P = 42 HC = 49	CBP	P = Mild HC = None	Trait	P = Minimal HC = Minimal	P = 6 D = NA	NR	Pain corr dep	Pos	ACC DLPFC	rs-fMRI	Y
Apkarian 2004 [76]	P = 26 HC = 26	P = 43 HC = 43	CBP	P = Moderate HC = NR	Trait	P = Mild HC = NR	P = 136 D = NA	NR	Pain corr dep	ns	ns	MRI	N
As-Sanie 2016 [41]	P = 36 HC = 37	P = 31 HC = 29	CPP	P = Moderate HC = Minimal	Trait	P = Mild HC = NR	P = 6 D = NA	Inc	Pain corr dep	Pos	Insula MPFC	rs-fMRI	Y
Bär 2007 [47]	D = 13 HC = 13	D = 36 HC = 34	Exp.	P = threshold at 44 °C HC = threshold at 41 °C	Trait	D = Moderate HC = Minimal	P = NA D = 97	Exc	Dep v HC	Dep > HC	L thalamus	task-fMRI	Y^a^
Berna 2010^53^	HC = 20	HC = 28	Exp.	P = Moderate HC = Moderate	Exp.	HC = Minimal	NA	NR	Dep v HC	Dep > HC Dep < HC	Superior parietal lobe Precuneus Supramarginal gyrus IFG S2 DLPFC ACC OFC Temporal gyrus Insula Hippocampus Thalamus Caudate Occipital Intraparietal sulcus Temporal gyrus B S1 Precuneus	task-fMRI	Y^a,b^
Bilek 2019 [77]	D = 37 HC = 33	D = 34 HC = 26	Exp.	D = Moderate HC = Moderate	Dx	D = NR HC = NR	P = NA D = NR	NR	Dep corr pain	Dep < HC	Frontal, temporal and occipital network	task-fMRI	N
Cifre 2012 [78]	P = 9 HC = 11	P = 52 HC = 49	FM	P = Severe HC = NR	Trait	P = Severe HC = Minimal	P = 322 D = NA	Inc	Pain v HC	Pain < HC	R Thalamus^a^ R PAG L ACC^a^ L PAG	rs-fMRI	Y
Coulombe 2017 [79]	P = 23 HC = 16	P = 51 HC = 50	FM	P = Moderate HC = NR	Trait	P = Minimal HC = NR	P = NR D = NA	Exc	Pain v HC	ns	ns	rs-fMRI	N
Diaz-Piedra 2016 [33]	P = 23 HC = 23	P = 42 HC = 40	FM	P = Moderate HC = Minimal	Trait	P = Moderate HC = Minimal	P = 103 D = NA	Inc	Pain v HC	ns	ns	MRI	N
Fan 2020 [27]	P = 42 HC = 35	P = 30 HC = 31	CD	P = Minimal HC = NR	Trait	P = Minimal HC = NR	P = 59 D = NA	NR	Pain corr dep	ns	ns	rs-fMRI	N
Feliu-Soler 2020 [48]	P = 47	P = 52	FM	P = Moderate	Trait	P = Mild	P = 145 D = 20 episodes	Inc	Pain corr dep	Pos	Bed nucleus of the stria terminalis	MRI	Y
Giesecke 2005 [35]	P = 53 HC = 42	P = 42 HC = 38	FM	P = Moderate HC = None	Trait	P = Mild HC = Minimal	P = NR D = NA	Exc	Pain corr dep	Neg	Insula B amygdala	task-fMRI	Y^a^
Grachev 2003 [43]	P + D = 10 HC = 10	P + D = 29 HC = 48	CBP	P + D = Moderate HC = NR	Trait	P + D = Mild HC = Minimal	P = 151 D = 114	Exc	Pain v HC	Pain < HC	R DLPFC	MRS	N
Guedj 2008 [80]	P = 20 HC = 10	P = 48 HC = 52	FM	P = Severe HC = None	Trait	P = Moderate HC = Minimal	P = NR D = NA	NR	Pain corr dep	ns	ns	SPECT	N
Gustin 2013 [39]	P = 42 HC = 35	P = 49 HC = 50	NeuP	P = Mild HC = NR	Trait	P = Mild HC = Minimal	P = 144 D = NA	NR	Pain corr dep	Neg (state dep) Pos (state dep) Neg (trait dep) Pos (trait dep)	B thalamus ACC DLPFC PCC B hippocampus B thalamus DLPFC PCC Insula B hippocampus	MRI	N
Harfeldt 2018 [28]	P = 36 HC = 10	P = 42 HC = 36	NeuP	P = Moderate HC = None	Trait	P = Moderate HC = Minimal	P = NR D = NA	Exc	Pain corr dep	ns	ns	MRS	N
Icenhour 2019 [81]	P = 64 HC = 32	P = 30 HC = 33	IBS	P = Moderate HC = Minimal	Trait	P = Minimal HC = Minimal	P = NR D = NA	NR	Pain corr dep	ns	ns	MRS	N
Ikeda 2018 [82]	P = 23 HC = 17	P = 48 HC = 42	Unspecified CP	P = Moderate HC = None	Trait	P = Mild HC = Minimal	P = 103 D = NA	NR	Pain corr dep	Neg	R insula^a^ L nucleus accumbens	rs-fMRI	Y
Ivo 2013 [83]	P = 14 HC = 14	P = 54 HC = NR	CBP	P = Severe HC = NR	Trait	P = Moderate HC = Minimal	P = 120 D = NA	NR	Pain v HC	ns	ns	MRI	N
Jensen 2013 [84]	P = 26 HC = 13	P = 38 HC = 34	FM	P = Moderate HC = NR	Trait	P = Moderate HC = NR	P = 132 D = NA	Exc	Pain corr dep	Neg	R percalcarine R fusiform R occipital L inferior parietal L caudal middle frontal L precuneus L fusiform L supra marginal	task-fMRI	Y^a^
Jin 2017 [85]	P = 33 HC = 32	P = 22 HC = 22	PD	P = Moderate HC = Minimal	Trait	P = Minimal HC = Minimal	P = 78 D = NA	NR	Pain corr dep	ns	ns	rs-fMRI	N
Kameda 2018 [86]	P = 34 HC = 56	P = 59 HC = 40	CBP	P = Moderate HC = NR	Trait	P = Minimal HC = NR	P = NR D = NA	Inc	Pain corr dep	Neg	ACC	MRS	N
Ke 2015 [87]	P = 31 HC = 32	P = 29 HC = 28	IBS	P = Moderate HC = NR	Trait	P = Minimal HC = Minimal	P = 33 D = NA	NR	Pain corr dep	ns	ns	rs-fMRI	N
Khan 2014 [36]	P = 9 HC = 9	P = 54 HC = 56	BMS	P = Minimal HC = NR	Trait	P = Minimal HC = Minimal	P = 48 D = NA	Exc	Pain corr dep Pain v HC	Pos Pain > HC	L MPFC^a^ R hippocampus L MPFC^a^ R Hippocampus	rs-fMRI	Y
Kim 2015 [40]	P = 42 HC = 63	P = 45 HC = 43	FM	P = Moderate HC = NR	Trait	P = Mild HC = Minimal	P = 167 D = NA	NR	Pain corr dep	Pos	Orbital frontal gyrus Gyrus rectus Superior medial frontal gyrus L lingual gyrus B supramarginal gyrus Fusiform gyrus B medial temporal lobe R inferior parietal	MRI	N
Klega & Eberle 2010 [88]	P = 10 HC = 10	P = 46 HC = NR	CRPS	P = Mild HC = None	Trait	P = Minimal HC = NR	P = 4 D = NA	NR	Pain corr dep	Pos	Temporal cortex	PET	Y
Ledermann 2016 [89]	P + D = 11 P = 13 HC = 17	P + D = 55 P = 45 HC = 43	FM	P + D = Mild P = Mild HC = Minimal	Trait	P + D = Mild P = Minimal HC = Minimal	P = 162 D = NA	Inc	Pain/Dep	P-D > P	L ventral striatum	PET	N
Lee 2015 [90]	P = 24 HC = 25	P = 36 HC = 32	CRPS	P = NR HC = NR	Trait	P = Moderate HC = Minimal	P = 34 D = NA	Inc	Pain v HC	ns	ns	MRI	N
J. Li 2020 [44]	P + D = 28 P = 21 HC = 36	P + D = 36 P = 32 HC = 32	IBS	P + D = Mild P = Minimal HC = NR	Trait	P + D = Mild P = Minimal HC = Minimal	P + D = 21 P = 19	Exc	Pain/Dep Pain v HC	P-D < P Pain < HC	B MFG L IFG L SFG L Insula L IFG	MRI	Y
H. Li 2020 [91]	P = 24 HC = 23	P = 64 HC = 61	NeuP	P = Moderate HC = None	Trait	P = Minimal HC = Minimal	P = 22 D = NA	NR	Pain corr dep	Neg	PAG^a^ S1	rs-fMRI	N
Lirng 2015 [92]	P + D = 16 P = 14	P + D = 41 P = 40	H/M	P + D = Severe P = Moderate	Trait	P + D = Moderate P = Minimal	P = 200 (P + D) P = 154 (P) D = NA (P + D) D = NA (P)	Inc	Pain/Dep	P-D > P	R DLPFC	MRS	N
P. Liu 2013 [93]	P = 49 HC = 39	P = 22 HC = 22	FD	P = Minimal HC = Minimal	Trait	P = Minimal HC = Minimal	P = 36 D = NA	Exc	Pain corr dep	ns	ns	rs-fMRI	N
B-L. Liu 2015 [94]	P = 29 HC = 10	P = 54 HC = 52	BMS	P = Mild HC = NR	Trait	P = Minimal HC = Minimal	P = 10 D = NA	NR	Pain/Dep Pain v HC	P-D < P Pain < HC	L Parietal L Temporal L Parieto-occipital lobe	SPECT	N
P. Liu 2017 [25]	P = 66 HC = 42	P = 23 HC = 23	FD	P = NR HC = NR	Trait	P = Minimal HC = Minimal	P = 35 D = NA	NR	Pain/Dep	ns	ns	rs-fMRI	N
P. Liu, Li, 2018 [26]	P = 43 HC = 37	P = 32 HC = 31	CD	P = NR HC = NR	Trait	P = Minimal HC = Minimal	P = 80 D = NA	NR	Pain/dep	ns	ns	rs-fMRI	N
P. Liu, Wang 2018 [95]	P = 69 HC = 49	P = 23 HC = 23	FD	P = Minimal HC = NR	Trait	P = Minimal HC = Minimal	P = NR D = NA	Exc	Pain corr dep	ns	ns	MRI	N
Q. Liu 2019 [34]	P = 21 HC = 17	P = 43 HC = 43	PSPD	P = Moderate HC = NR	Trait	P = Moderate HC = Minimal	P = 42 D = NA	Exc	Pain v HC	ns	ns	SPECT	N
López-Solà 2017 [96]	P = 37 HC = 35	P = 46 HC = 44	FM	P = Moderate HC = NR	Trait	P = Mild HC = NR	P = 80 D = NA	Inc	Pain corr dep	Pos	ACC Precuneus	task-fMRI	N
Luo 2016 [97]	P = 12 HC = 10	P = 46 HC = 35	PSPD	P = Moderate HC = None	Trait	P = Minimal HC = Minimal	P = 6 D = NA	Exc	Pain/Dep	P-D > D	L Fusiform gyrus B DLPFC L VLPFC MPFC	task-fMRI	N
Ma 2018 [98]	P + D = 10 P = 22 HC = 27	P + D = 28 P = 34 HC = 30	H/M	P = Severe HC = NR	Trait	P + D = Moderate P = Minimal HC = Minimal	P = 86 (P + D) P = 118 (P) D = NA (P + D) D = NA (P)	NR	Pain/Dep	P-D < P P-D > P	Thalamus Fusiform	rs-fMRI	Y
Malejko 2021 [45]	D = 22 HC = 25	D = 23 HC = 20	Exp.	Not compared	Dx	D = Severe HC = Minimal	P = NA D = NA	Inc	Dep corr pain	Neg	R S1	task-fMRI	Y^a^
Mao & Yang 2015 [99]	P = 33 HC = 33	P = 51 HC = 51	CBP	P = Moderate HC = NR	Trait	P = Minimal HC = Minimal	P = 114 D = NA	Exc	Pain corr dep	ns	ns	MRI	N
Mountz 1995 [100]	P = 10	P = 44	FM	P = Mild	Trait	P = Mild	P = NR D = NA	Exc	Pain corr dep	ns	ns	SPECT	N
Nees 2019 [101]	P = 39 HC = 30	P = 45 HC = 41	CBP	P = Mild HC = Minimal	Trait	P = Mild HC = Minimal	P = 104 D = NA	Inc	Pain corr dep	ns	ns	Cortisol	N
Niddam 2018 [102]	P = 49 HC = 25	P = 35 HC = 33	H/M	P = Moderate HC = NR	Trait	P = Minimal HC = Minimal	P = 160 D = NA	Exc	Pain corr dep	Neg	ACC	MRS	N
Otti 2013 [103]	P = 21 HC = 19	P = 47 HC = 49	SomP	P = Moderate HC = NR	Trait	P = Mild HC = Minimal	P = NR D = NA	Inc	Pain corr dep	ns	ns	rs-fMRI	N
Qi 2016 [29]	P = 65 HC = 67	P = 34 HC = 31	IBS	P = Mild HC = NR	Trait	P = Minimal HC = Minimal	P = 42 D = NA	Exc	Pain corr dep	ns	ns	rs-fMRI	N
Robinson 2011 [104]	P = 14 HC = 11	P = 43 HC = 42	FM	P = MildHC = NR	Trait	P = Minimal HC = Minimal	P = NR D = NA	NR	Pain corr dep	ns	ns	MRI	N
Rosenberger 2013 [105]	P = 15 HC = 12	P = 42 HC = 31	IBS	P = Moderate HC = Mild	Trait	P = Minimal HC = Minimal	P = 12 D = NA	Exc	Pain corr dep	Pos	Cerebellum	task-fMRI	Y^a^
Schweinhardt 2008 [42]	P = 20	P = 57	RA	P = Moderate	Trait	P = Minimal	P = NR D = NA	Exc	Pain corr dep	Pos	MPFC	task-fMRI	Y^a^
Seifert 2011 [106]	P = 13	P = 44	H/M	P = Mild HC = NR	Trait	P = Mild	P = 2 D = NA	Inc	Pain corr dep	Pos	Insula	rs-fMRI	Y
Smit 2018 [30]	P = 14 HC = 12	P = 56 HC = 53	Cervical dystonia	P = Mild HC = NR	Trait	P = Minimal HC = Minimal	P = 144 D = NA	Exc	Pain corr dep	ns	ns	PET	N
Strigo 2008 [46]	D = 15 HC = 15	D = 25 HC = 24	Exp.	P = NR HC = NR	Dx	D = Moderate HC = NR	P = NA D = NA	Exc	Dep corr pain	Dep > HC Dep < HC	R amygdala L parahippocampal gyrus Visual cortexR ACC R DLPFC L DLPFC L SFG L MTG L precuneus R PAG R brainstem	task-fMRI	Y^a^
Tan 2019 [31]	P = 26 HC = 27	P = 52 HC = 51	BMS	P = MildHC = NR	Trait	P = Minimal HC = Minimal	P = 9 D = NA	NR	Pain corr dep	ns	ns	MRI	N
Truini 2016 [107]	P = 20 HC = 15	P = 28–67 range HC = 26–65 range	FM	P = Moderate HC = NR	Trait	P = Minimal HC = NR	P = 93 D = NA	Exc	Pain corr dep	Neg	PAG^a^ Opercular cortex SFG Supramarginal gyrus	rs-fMRI	N
Vachon-Presseau 2016 [37]	P = 69 HC = 20	P = 43 HC = 37	BP	P = Moderate HC = NR	Trait	P = Minimal HC = NR	P = NR D = NR	Inc	Pain corr dep	Pos	Amygdala Nucleus accumbens MPFC	rs-fMRI	Y
Valdes 2010 [108]	P = 28 HC = 24	P = 43 HC = 44	FM	P = Moderate HC = NR	Trait	P = Mild HC = Minimal	P = 151 D = NA	NR	Pain corr dep	ns	ns	MRS	N
van Ettinger-Veenstra 2019 [109]	P = 38 HC = 37	P = 46 HC = 51	Unspecified CP	P = Moderate HC = None	Trait	P = Mild HC = Minimal	P = NR D = NA	Inc	Pain corr dep	ns	ns	rs-fMRI	N
Wagner 2009 [110]	HC = 40	HC = 25	Exp.	HC = Pain sensitivity +	Exp.	HC = NR	P = NA D = NA	NR	Pain/Dep	P-D > P	B Thalamus	task-fMRI	Y^a,b^
Y. Wang 2017 [111]	P = 38 HC = 38	P = 56 HC = 56	NeuP	P = NR HC = NR	Trait	P = Minimal HC = Minimal	P = 85 D = NA	Exc	Pain corr dep	Neg	Insula^a^ ACC	rs-fMRI	Y
D. Wang 2017 [32]	P = 31 HC = 30	P = 44 HC = 43	IBS	P = Moderate HC = NR	Trait	P = Minimal HC = Minimal	P = 88 D = NA	NR	Pain corr dep	ns	ns	task-fMRI	N
Wingenfeld 2010 [112]	P = 21 HC = 26	P = 33 HC = 35	CPP	P = Minimal HC = Minimal	Trait	P = Mild HC = NR	P = 21 D = NA	Inc	Pain/Dep	P-D < P	HPA	Cortisol	N
Woldeamanuel 2019 [113]	P = 30 HC = 30	P = 40 HC = 40	H/M	P = Moderate HC = NR	Trait	P = Mild HC = Minimal	P = 312 D = NA	Inc	Pain corr dep	ns	ns	MRI	N
Q. Yang 2017 [114]	P = 49 HC = 49	P = 51 HC = 55	H/M, CBP	P = Moderate HC = NR	Trait	P = Minimal HC = Minimal	P = 168 D = NA	Inc	Pain corr dep	ns	ns	rs-fMRI	N
Yu 2013 [115]	P = 40 HC = 40	P = 36 HC = 33	H/M	P = Moderate HC = NR	Trait	P = Moderate HC = Minimal	P = 130 D = NA	Exc	Pain/Dep	P-D < P	Caudate	rs-fMRI	N
Zhang 2018 [38]	P = 29 HC = 34	P = 48 HC = 43	NeuP	P = Moderate HC = NR	Trait	P = Minimal HC = None	P = 72 D = NA	Exc	Pain corr dep	Pos	R amygdala^a^ Medial frontal gyrus	rs-fMRI	N
Zunhammer 2016 [116]	HC = 39	HC = 26	Exp.	HC = Pain sensitivity +	Trait	HC = Minimal	P = NA D = NA	Inc	Pain corr dep	Pos	ACC Insula	MRS	N
*Details of 2 additional studies (3 analyses) (August 2020–September 2021 search) included in the systematic review*													
Hou 2021 [117]	HC = 25 P + D = 17 D = 19	HC = 23 P + D = 23 D = 22	Exp.	HC = Moderate P + D = Moderate D = Moderate	Dx	HC = Normal P + D = Moderate D = Moderate	P + D (D) = 17 D (D) = 14	Exc	Dep v HC Pain/Dep	D < HC P-D > D	L Rolandic operculum R temporal lobe R fusiform gyrus R insula L PCG L parietal inferior gyrus L temporal lobe	task-fMRI	Y^a^
Kim 2020 [118]	HC = 18 P = 28	HC = 46 P = 41	Chronic musculoskeletal + FM	HC = Normal P = Mild	Trait	HC = Normal D = Normal-mild	P = NR D = NR	NR	Pain/Dep v HC	ns	ns	task-fMRI, PET	N

*BMS* Burning mouth syndrome, *BP* Back pain, *CBP* Chronic back pain, *CD* Crohn’s disease, *CPP* Chronic pelvic pain, *CRPS* Complex regional pain syndrome, *D* Depression sample, *Dx* Diagnosis, *Exc* Excluded at time of study, *Exp* Experimentally induced, *FD* Functional dyspepsia, *FM* Fibromyalgia, *H/M* Headache/migraine, *HC* Healthy control, *Inc* Included at time of study, *IBS* irritable bowel syndrome, *NA* Not applicable, *NR* Not reported, *NeuP* Neuropathic pain, *P* Pain sample, *P* + *D* Simultaneous pain and depression, *PD* Primary dysmenorrhea, *PCG* postcentral gyrus, *PSPD* Persistent somatic pain disorder, *RA* Rheumatoid arthritis, *SomP* Somatic pain disorder, *Unspecified CP* Unspecified chronic pain.

Comparison types:

*Pain/Dep* Simultaneous pain and depression comorbidity studies, *Pain corr dep* Pain correlated with concurrent depression, *Pain v HC* Pain contrasted with HC, with depression as covariate, *Dep corr pain* Depression correlated with concurrent pain, *Dep v HC* Depression contrasted with HC, with pain as covariate, or in pain-related brain activation; *ns* non-significant.

Neuroimaging:

*task-fMRI* task functional magnetic resonance imaging, *PET* positron emission tomography, *MRI* magnetic resonance imaging, *SPECT* single-photon emission computed tomography, *MRS* magnetic resonance spectroscopy, *rs-fMRI* resting-state functional magnetic resonance imaging.

ALE legends:

*N* Study not included in ALE analysis, *Y* Study included in ALE analysis, ^a^Experimentally induced pain, ^b^Experimentally induced depression/sad mood

#### Measures of pain

Pain was measured by visual analogue scale (VAS), McGill Pain Questionnaire (standard and short-form), numerical rating scale, Brief Pain Inventory, West Haven-Yale Multidimensional Pain Inventory (WHYMPI), Fibromyalgia Impact Questionnaire (FIQ), Crohn’s Disease Endoscopic Index of Severity (CDEIS), Crohn’s Disease Activity Index (CDAI), Tubingen Pain Behaviour Scale, Questionnaire Douleur de Saint-Antoine Scale, IBS Severity Scoring System (IBS-SSS), PainDETECT Questionnaire, Oswestry Disability Index (ODI), Cox Retrospective Symptom Scale, Pain Disability Assessment Scale (PDAS), Gastrointestinal Symptoms Rating Scale (GSRS), Present Pain Intensity (PPI), Nepean Dyspepsia Index (NDI), Inflammatory Bowel Disease Questionnaire (IBDQ), Headache Impact Test (HIT-6), Migraine Disability Assessment Questionnaire (MIDAS), Medical College of Virginia Pain Questionnaire, Disease Activity Score (DAS28), Toronto Western Spasmodic Torticollis Rating Scale, Pain Experience Scale (PES), Patient Health Questionnaire-15 (PHQ-15).

#### Measures of depression

Depression was measured by Beck’s Depression Inventory (BDI-II), State-Trait Personality Inventory (STPI-D), Hamilton Depression Rating Scale (HDRS), Hospital Anxiety and Depression Scale-Depression (HADS-D), Centre for Epidemiologic Studies Depression Scale (CES-D), Patient Health Questionnaire (PHQ-9), Zung Self-Rating Depression Scale (SDS), Montgomery Asberg Depression Sale (MADRS), and VAS.

#### Types and severity of pain

The main sample (68 studies) consisted of participants presenting with a range of pain types: chronic back pain 10%, chronic pelvic pain 1%, fibromyalgia 22%, Crohn’s disease 3%, irritable bowel syndrome 8%, primary dysmenorrhea 1%, burning mouth syndrome 4%, complex regional pain syndrome 3%, neuropathic pain 7%, headache/migraine 10%, functional dyspepsia 4%, somatic pain disorder 4%, rheumatoid arthritis 1%, cervical dystonia 1%, unspecified chronic pain 3% and experimentally induced pain 11%. Out of the 54 pain analyses, five reported *minimal pain* on a group-level average (9%), 12 reported *mild pain* (22%), 31 reported *moderate pain* (57%), three reported *severe pain* (6%) and three did not reported classifiable pain severity (6%). Out of the five primary-depression analyses, two reported *moderate pain* on a group-level average (60%) and three did not report classifiable pain severity (60%). Out of the 12 pain and depression comorbidity analyses, four (33%) reported *minimal* to *mild* pain, five (42%) reported *moderate* to *severe* pain, and three did not report classifiable pain severity. The 2 studies identified in the 2020–2021 search reported mild and moderate pain severity for the chronic musculoskeletal and fibromyalgia patients and induced pain in healthy controls respectively.

#### Types and severity of depression

In terms of depression, 93% of the main sample (68 studies) were assessed on baseline depressive traits, 4% were diagnosed with Major Depressive Disorder (MDD), and 3% were subjected to experimentally induced sadness. Out of the 54 pain analyses, 29 reported *minimal depression* (54%), 17 reported *mild depression* (31%), seven reported *moderate depression* (13%), 1 sample reported *severe depression* (2%). Out of the five depression analyses, one reported *minimal depression* (20%), three reported *moderate* to *severe* depression (60%), and one (20%) did not report classifiable severity. The 2 studies identified in the 2020-2021 search reported moderate and normal to mild depression levels.

Out of the 12 pain and depression comorbidity analyses in the main sample (68 studies), five (42%) reported minimal depression, three (25%) reported mild depression, three (25%) reported moderate depression and one (8%) did not report classifiable severity of depression. The additional study identified in the 2020-2021 search reported moderate severity for the depression with pain group.

#### Neuroimaging

Of the 13 pain and depression comorbidity analyses in all 70 studies, four were task functional magnetic resonance imaging (task-fMRI) where participants were exposed to either induced pain or sadness, four were resting-state fMRI (rs-fMRI), one of PET, MRI, SPECT, MRS, and cortisol profiling each. Out of the 54 pain analyses, seven (13%) were task-fMRI studies, 21 (39%) were rs-fMRI studies, two (4%) were positron emission tomography (PET) studies, three (6%) were single-photon emission computed tomography (SPECT) studies, 12 (22%) were structural MRI-grey matter (MRI-GM) studies, seven (13%) were magnetic resonance spectroscopy (MRS) studies, one (2%) was a computed tomography (CT) study, one (2%) used diurnal cortisol profiling. All six depression analyses used task-fMRI.

#### Study quality assessment

All 70 papers clearly stated study aim and defined population of interest (Table 2). The majority of the studies did not report size of eligible population, it was therefore unclear whether sampling was in any way biased. Another area of weakness was the lack of statistical justification for the chosen sample size, or provision of variance estimates. Positively, both exposure and outcome variables were clearly defined and consistently implemented across all study participants. Where appropriate, qualification of item 7 requires that the study sample consisted of patients with disease duration greater than one year. Given that the aim of the current project was to collate neuroimaging findings (typically found in cross-sectional studies), the absence of repeated assessments of the exposure variable or follow-up measurement were not considered detrimental to study quality.

**Table 2:** Study quality assessment.

	Study quality assessment with the National Institutes of Health Quality Assessment Tool.															
Item		1	2	3	4	5	6	7	8	9	10	11	12	13	14	Overall
Adler-Neal et al. 2019 [74]		√	√	√	√	√	√	---	√	√	---	√	---	---	√	Good
Albrecht et al. 2019 [75]		√	√	--	-	√	√	-	√	√	---	√	--	---	--	Fair
Apkarian et al. 2004 [76]		√	√	--	√	√	√	√	√	√	---	√	--	---	√	Good
As-Sanie et al. 2016 [41]		√	√	--	-	×	√	√	√	√	---	√	√	---	√	Fair
Bar et al. 2007 [47]		√	√	--	√	√	√	√	-	√	---	√	--	---	√	Good
Berna et al. 2010 [53]		√	√	√	√	√	√	---	√	√	---	√	---	---	---	Good
Bilek et al. 2019 [77]		√	√	--	√	×	√	--	-	√	---	√	--	---	√	Fair
Cifre et al. 2012 [78]		√	√	--	-	√	√	√	×	√	---	√	--	---	×	Fair
Coulombe et al. 2017 [79]		√	√	--	√	---	√	--	√	√	---	√	--	---	-	Fair
Diaz-Piedra et al. 2016 [33]		√	√	√	√	√	√	√	×	√	---	√	--	---	√	Good
Fan et al. 2020 [27]		√	√	√	√	×	--	√	×	√	---	√	--	---	-	Fair
Feliu-Soler et al. 2020 [48]		√	√	--	√	√	√	√	×	√	---	√	--	---	--	Poor
Giesecke et al. 2005 [35]		√	√	--	√	√	√	--	√	√	---	√	--	---	√	Good
Grachev et al. 2003 [43]		√	√	--	-	√	√	√	×	√	---	√	--	---	√	Fair
Guedj et al. 2008 [80]		√	√	--	√	---	--	--	√	√	---	√	--	---	√	Good
Gustin et al. 2013 [39]		√	√	--	-	√	√	√	×	√	---	√	--	---	×	Fair
Harfeldt et al. 2018 [28]		√	√	--	√	×	√	-	×	√	---	√	--	---	-	Poor
Icenhour et al. 2019 [81]		√	√	--	-	√	√	--	×	√	---	√	--	---	√	Fair
Ikeda et al. 2018 [82]		√	√	√	√	√	√	√	×	√	---	√	--	---	√	Good
Ivo et al. 2013 [83]		√	√	--	-	---	√	√	×	√	---	√	--	---	--	Fair
Jensen et al. 2013 [84]		√	√	×	-	×	√	√	√	√	---	√	--	---	√	Fair
Jin et al. 2017 [85]		√	√	--	√	×	√	√	×	√	---	√	--	---	--	Poor
Kameda et al. 2018 [86]		√	√	√	-	√	√	-	×	√	---	√	--	---	-	Fair
Ke et al. 2015 [87]		√	√	--	-	---	√	√	×	√	---	√	--	---	--	Fair
Khan et al. 2014 [36]		√	√	--	√	√	√	√	-	√	---	√	--	---	√	Good
Kim et al. 2015 [40]		√	√	--	-	√	√	√	×	√	---	√	--	---	--	Fair
Klega et al. 2010 [88]		√	√	--	√	√	√	×	√	√	---	√	--	---	√	Good
Ledermann et al. 2016 [89]		√	√	--	√	---	√	√	√	√	---	√	--	---	√	Good
Lee et al. 2015 [90]		√	√	--	√	---	√	-	×	√	---	√	--	---	√	Fair
Li H et al. 2020 [91]		√	√	√	√	√	√	-	×	√	---	√	--	---	√	Good
Li J et al. 2020 [44]		√	√	√	√	√	√	√	√	√	---	√	--	---	--	Good
Lirng et al. 2015 [92]		√	√	--	√	√	√	√	-	√	---	√	√	---	--	Good
Liu et al. 2013 [93]		√	√	√	√	---	√	√	×	√	---	√	--	---	√	Good
Liu et al. 2015 [94]		√	√	--	-	×	√	×	√	√	---	√	--	---	√	Poor
Liu, Liu, Wang et al. 2017 [25]		√	√	--	√	---	√	√	×	√	---	√	--	---	--	Fair
Liu, Wang, Liu et al. 2017 [26]		√	√	--	√	×	√	√	×	√	---	√	--	---	√	Fair
Liu et al. 2019 [34]		√	√	--	√	√	√	√	×	√	---	√	--	---	√	Good
Liu et al. 2018 [95]		√	√	--	√	×	√	√	×	√	---	√	√	---	--	Fair
Lopez-Sola et al. 2017 [96]		√	√	√	√	×	√	√	×	√	---	√	--	---	√	Fair
Luo et al. 2016 [97]		√	√	--	√	×	√	-	√	√	---	√	--	---	√	Poor
Ma et al. 2018 [98]		√	√	√	√	×	√	√	×	√	---	√	--	---	-	Fair
Malejko et al. 2021 [45]		√	√	√	√	√	√	--	×	√	---	√	--	---	√	Good
Mao et al. 2015 [99]		√	√	--	-	---	√	√	×	√	---	√	--	---	√	Fair
Mountz et al. 1995 [100]		√	√	--	√	×	√	--	×	√	---	√	--	---	√	Poor
Nees et al. 2019 [101]		√	√	--	√	---	--	√	×	√	---	√	--	---	√	Fair
Niddam et al. 2018 [102]		√	√	--	√	√	√	--	×	√	---	√	--	---	--	Fair
Otti et al. 2013 [103]		√	√	-	-	---	√	--	×	√	---	√	--	---	-	Poor
Qi et al. 2016 [29]		√	√	--	√	×	√	√	×	√	---	√	--	---	√	Fair
Robinson et al. 2011 [104]		√	√	--	√	√	√	--	×	√	---	√	√	---	×	Good
Rosenberger et al. 2013 [105]		√	√	--	√	×	√	√	×	√	---	√	--	---	√	Fair
Schweinhardt et al. 2008 [42]		√	√	--	√	---	√	--	×	√	---	√	--	---	√	Fair
Seifert et al. 2011 [106]		√	√	--	-	×	√	-	×	√	---	√	--	---	√	Poor
Smit et al. 2018 [30]		√	√	√	√	√	√	√	×	√	---	√	--	---	√	Good
Strigo et al. 2008 [46]		√	√	--	√	×	√	√	×	√	---	√	--	---	√	Fair
Tan et al. 2019 [31]		√	√	--	√	√	√	×	×	√	---	√	--	---	-	Poor
Truini et al. 2016 [107]		√	√	--	-	√	√	√	×	√	---	√	--	---	√	Fair
Vachon-Presseau et al. 2016 [37]		√	√	×	×	-	√	×	×	√	√	√	--	×	√	Poor
Valdes et al. 2010 [108]		√	√	√	√	-	√	√	×	√	---	√	--	---	--	Fair
van Ettinger-Veenstra et al. 2019 [109]		√	√	√	√	√	√	--	×	√	---	√	--	---	√	Good
Wagner et al. 2009 [110]		√	√	√	√	×	√	---	×	√	---	√	--	---	√	Fair
Wang et al. 2017 [32]		√	√	√	√	×	√	√	×	√	---	√	--	---	√	Fair
Wang Y et al. 2017 [111]		√	√	--	-	√	√	√	×	√	---	√	√	---	--	Fair
Wingenfeld et al. 2017 [112]		√	√	√	√	×	√	√	√	×	---	√	--	---	√	Poor
Woldeamanuel et al. 2019 [113]		√	√	--	-	×	√	--	×	√	---	√	--	---	×	Poor
Yang et al.2017 [114]		√	√	--	√	√	√	√	√	×	---	√	--	---	-	Fair
Yu et al. 2013 [115]		√	√	--	√	√	√	√	√	√	---	√	--	---	--	Good
Zhang et al. 2018 [38]		√	√	--	√	×	√	√	×	√	√	√	--	×	√	Fair
Zunhammer et al. 2016 [116]		√	√	--	√	√	√	---	×	√	---	√	--	---	√	Good
*Additionally identified studies*																
Hou 2021 [117]		√	√	--	√	×	√	√	√	√	---	√	--	---	√	Fair
Kim 2020 [118]		√	√	--	√	√	√	-	√	√	---	√	--	---	×	Fair

√ represents yes, × represents no, - represents could not determine, -- represents not reported, --- represents not applicable.

Scoring system: 1 point for each item that has ‘non-applicable’, ‘not reported’, ‘could not determine’ (due to either uncertainty or partial fulfillment of item requirement), or ‘no’ (failing to fulfil item requirement); further 0.5 point for failure to justify sample size or provide effect size/variance measures. Total numerical score is the sum of all points. Overall qualitative ratings are attributed based on the following system: ‘Good’ corresponds to the score of ≤3 and ‘no’=0; ‘Fair’ corresponds to the score ≥3.5 and ≤5; ‘Poor’ corresponds to the score ≥5.5.

### Analysis of pain-depression comorbidity studies (equal pain and depression symptoms)

Out of the 13 analyses which examined pain-depression comorbidity, 11 reported a significant association, 2 studies measured depression in combination with anxiety and were thus excluded [25, 26]. Of the 11 studies, 2 compared pain-depression comorbidity to depression [27, 28], and 9 to pain. In summary, task-fMRI data identified the PFC, fusiform gyrus, thalamus, and the insula as showing increased neural activation in comorbid moderate pain and minimal depression [28–30]. Participants who reported co-occurring mild pain and depression showed a decrease in grey matter volume in the DLPFC and the insula [31]. Participants with co-occurring moderate to severe pain and moderate depression showed lower resting-state activity in the thalamus and caudate [32, 33].

### Analysis of studies reporting pain or depression as a primary diagnosis

#### Primary pain with depression

Out of the 54 analyses, 19 did not report significant pain-depression correlations. Eight other studies were missing relevant data [27–34], such as depression measured in combination with anxiety and correlational results not reported. Of the remaining 27 analyses reporting significant results, the anterior cingulate cortex (ACC), posterior cingulate cortex (PCC), prefrontal cortex (PFC), insula, hippocampus/amygdala, caudate, thalamus and subregions of the temporal lobes and cerebellum were most frequently identified as key brain regions.

The amygdala/hippocampus were both identified as regions in which minimal to moderate pain and minimal to mild depression showed a positive correlation in a task-fMRI study [35], rs-fMRI studies [36–38], and structural MRI studies of grey matter [39, 40]. One structural MRI study [39] identified the thalamus as a region where mild pain and depression were negatively correlated. The MPFC also showed a positive correlation in rs-fMRI studies [37, 41] and a task-fMRI study [42]. In both cases, most participants presented with moderate levels of pain severity, and on average reported minimal to mild depressive traits. Data on the ACC and insula was less consistent across studies due to methodological heterogeneity.

No consistent pattern was identified out of the five studies that compared pain participants and healthy controls adjusting for depression, except that the DLPFC showed decreased activity in both MRS [43] and structural MRI [44] studies in participants with mild to moderate pain and mild depression.

#### Primary depression with pain

All six analyses found significant associations between depression and pain, two of which sampled patients clinically diagnosed with major depressive disorder [45, 46]. Malejko et al. [45] was the only task-fMRI study that identified the primary somatosensory cortex (S1) as negatively correlated in depression with induced pain; participants reported severe clinically diagnosed depression, but pain severity was unknown. Berna et al. (2010) and Strigo et al. (2008) identified hippocampus/amygdala using task-fMRI in participants with moderate pain and minimal to clinically significant depression (greater activity in depression/pain than in healthy controls), after adjusting ratings of induced pain. Findings about the involvement of the superior frontal gyrus (SFG), DLPFC, ACC and temporal gyri appeared less consistent. Task fMRI studies by [47] and Berna et al. (2010) both identified the thalamus as showing greater activation in depression than in healthy controls, adjusting for pain scores; both studies induced experimental pain in participants reporting minimal to moderate depressive traits.

### ALE meta-analysis

**Fig. 2:**
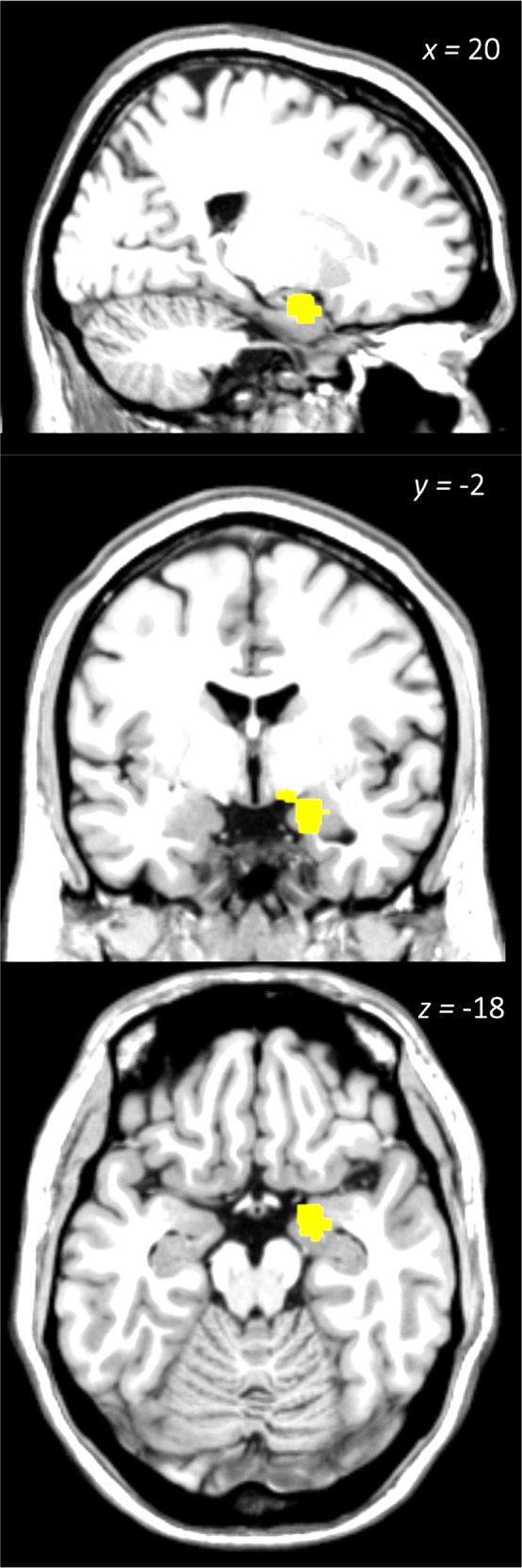
Neural correlate of pain with concomitant depression. Right parahippocampal gyrus/amygdala significantly associated with depression scores in pain samples.

**Fig. 3:**
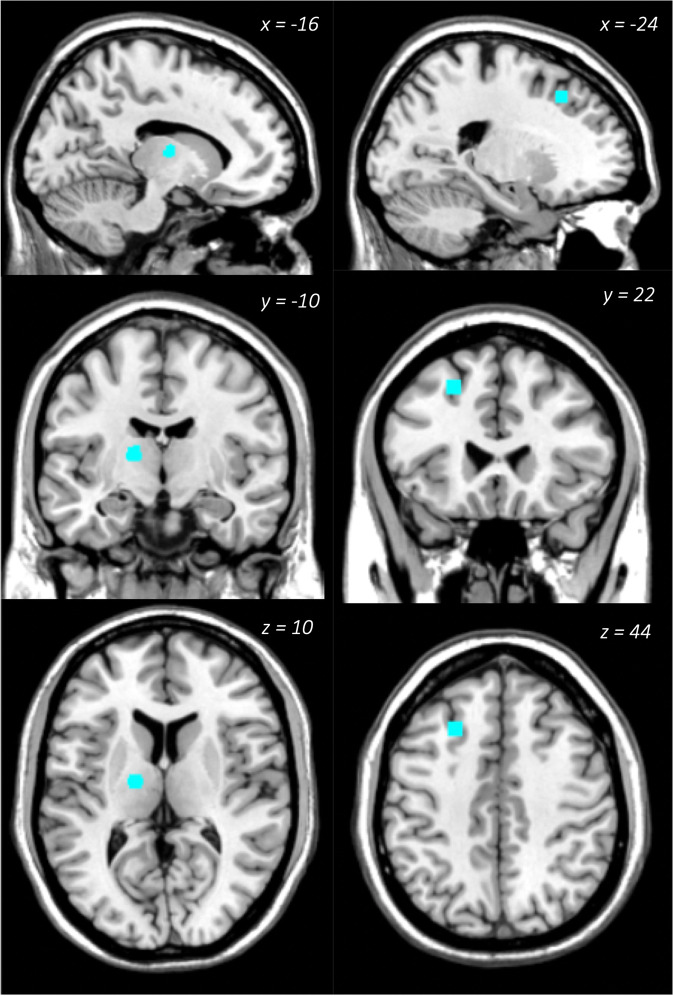
Neural correlates of depression with concomitant pain. Left superior frontal gyrus/DLPFC and left thalamus significantly associated with pain scores in depression samples.

Twenty-five of the eligible analyses in the original sample (68 studies) reported stereotaxic coordinates of significant results (see Table 1 for the information on the inclusion in ALE analysis). We analysed a total of 76 foci (20 studies, 586 participants) in studies of pain with reported depression scores, and 39 foci (5 studies, 119 participants) in studies of depression with reported pain scores (see Table 3). The ALE analysis identified the right parahippocampal gyrus/amygdala (*p* = 4.21E-07, *Z* = 4.92) as a neural correlate of pain associated with depression (Fig. 2), data contributed by five studies [35–38, 48] – all were studies of pain correlated with depression. Both the left superior frontal gyrus (cluster 1, *p* = 3.59E-07, *Z* = 4.96) and the left thalamus (cluster 2, *p* = 8.51E-07, *Z* = 4.78) were also identified as neural correlates of depression associated with pain (Fig. 3), data contributed by four studies (Berna et al., 2010; Strigo et al., 2008 for cluster 1; [47, 49] for cluster 2 – all were studies of depression versus healthy controls contrast with pain scores adjusted.

**Table 3:** Significant clusters and within-cluster peaks identified in global ALE analyses of both pain and depression as primary condition, presented separately for the original sample of 68 studies.

Label	Cluster size mm^3^	Coordinates (MNI)			*N* foci contrib.
		*x*	*y*	*z*	
Global analysis for pain as primary condition (76 foci, 586 subjects)					
Right parahippocampal gyrus/amygdala	1136	20	−2	−18	5
Global analysis for depression as primary condition (39 foci, 119 subjects)					
Left superior frontal gyrus	456	−24	22	44	2
Left thalamus^a^	424	−16	−10	10	2

^a^The left thalamus cluster was not observed when 2 additional studies identified in the 2020–2021 search were added.

When the ALE analysis in the Primary Depression contrast was repeated, including the additional 2 analyses identified in the updated 2020-2021 search, with 6 studies, 45 foci and 198 subjects included, we only observed the DLPFC cluster (cluster size, centre coordinate, and contributing foci unchanged from the previously reported analysis) but no significant cluster in the thalamus.

## Discussion

The aim of this review was to evaluate structural or functional brain alterations underlying comorbid pain and depression. Our literature search revealed that of the originally identified 68 neuroimaging studies (71 analyses) that reported measuring both pain and depression, only 17% (12 analyses) focused specifically on pain-depression comorbidity – no brain regions significantly associated with this contrast were identified by ALE. Only 1 additional study was identified when the literature search was extended to the period of August 2020–September 2021. The largest portion of the reviewed papers described the neurobiology of various pain conditions, finding a significant effect of co-occurring symptoms of depression on the underlying brain structure and function in at least 27 studies of those studies. In contrast, only 8.5% (6 analyses) of depression studies explored the link with pain. Although there is typically little difference made between pain with depression and depression with pain, using the ALE analysis, we were able to observe a distinction in the brain regions associated with depression in pain, namely the right amygdala region, and regions associated with pain in depression, specifically the left DLPFC and the thalamus. This highlights the possibility that the two types of comorbid pain and depression might involve distinct neural change that would necessitate differential treatments.

### Brain regions associated with pain and concomitant depression

Our ALE meta-analysis highlighted the role of the amygdala as a key region associated with depression in (chronic) pain conditions. Increased activation in the amygdala and paralimbic structures has been previously linked to the affective consequences of endogenous pain, potentially due to its structural and functional association with the neuropathology of emotion regulation and mood disorders [50].

Amygdala circuits have been specifically associated with pain-depression comorbidity, such as the one identified by Zhou and colleagues (2019) in mice. The pathway consists of 5-hydroxytryptamine (5-HT) projections from the dorsal raphe nucleus to somatostatin (SOM)-expressing and non-SOM interneurons in the central nucleus of the amygdala, which continue to extend directly towards the lateral habenula, an area known to be involved in depression. As a follow-up study testing the model in humans, the authors demonstrated a decrease in resting amygdala connectivity with serotonergic projections in chronic pain patients with comorbid depressive symptoms. This decrease in amygdala connectivity was not observed in depressed patients without chronic pain or chronic pain patients without depression, suggesting specificity of the amygdala connectivity with pain-depression comorbidity, consistent with our review findings.

Although the ALE analysis only revealed the amygdala/hippocampus cluster, our qualitative review of the pain with depression studies also consistently implicated MPFC in pain with depression, in line with existing literature [51–54]. Previous studies have demonstrated that neural representations of pain shifts from sensory to affective-related circuitry as the condition becomes chronic [55], highlighting the growing relevance of emotional difficulties commonly observed in chronic pain patients [55–57]. A recent review identified the MPFC-hippocampus circuit as critical in the development of depression in chronic clinical pain [58]. Consistent with this, another study [36] also reported a correlation between depression and altered MPFC-hippocampus connectivity in patients reporting high severity burning mouth syndrome, highlighting the impact of depressive symptoms on pain-related brain dysfunction in chronic burning mouth pain.

In relation to this, altered MPFC-amygdala connectivity has also been previously related to depression in pain. More specifically, recent studies have associated amygdala with the role of orienting the person’s attention towards salient stimuli [59], while the MPFC formulates an appraisal of the sensory experience by incorporating past experiences of persisting pain [60–62]. In line with this framework, Zhang and colleagues (2018) highlighted enhanced MPFC-amygdala functional connectivity in chronic trigeminal neuralgia patients, which was also correlated with depression. Furthermore another study [37] also reported dorsal MPFC-amygdala-nucleus accumbens network white matter density mediating the indirect effect of depression on pain chronification.

In summary, our data are in keeping with the literature, highlighting the relevance of the amygdala (and its adjacent limbic structures such as the hippocampus) for pain-associated negative mood in chronic pain. Of note is also the connectivity between MPFC and these limbic structures.

### Brain regions associated with depression and concomitant pain

Although there was a much smaller number of studies reporting depression with comorbid pain, there was a consistent association between depression with pain (5 of 6 studies), with the underlying brain regions converging on the thalamus and the DLPFC in our original ALE analysis.

The thalamus is commonly recognised as the ‘relay system’ from ascending afferent signals to cortical structures involved in higher level regulatory processes [63], and generally considered as a part of the afferent nociceptive network [64]. In line with the literature, our ALE-analysis identified the thalamus as significantly associated with pain in depressed patients. Both Berna et al. (2010) and Bär et al. (2007) reported an increase of thalamic activity in depressed patients undergoing painful stimulation. More specifically Bär et al. (2007) also reported a significant correlation between depression severity and thalamic activity. Given the previous research which suggested that thalamic activity could reflect attentional processing of sensory pain [65], the authors argued that depression is associated with increased attention to pain-related stimuli when afferent input is conveyed to the cortex, such as the DLPFC [47]. Note, however, that the thalamic cluster was not very robust, as the inclusion of only 2 additional analyses (from the 2020-2021 literature search) resulted in a non-significant result for the thalamic cluster. The DLPFC cluster remained significant in the reanalysis.

The DLPFC is responsible for both cognitive reappraisal and emotion regulation [66, 67]. The finding that the DLPFC is associated with the primary diagnosis of depression is not surprising. The DLPFC, specifically the left DLPFC, has long been recognised as a key area of depression pathophysiology [68]. Its role in pain and pain modulation [69–71] is also well established, including that it may be involved in pain suppression [72], cognitive and emotion control [73]. In line with these studies, greater left DLPFC activation correlated with smaller differences in pain unpleasantness between depressed- and neutral moods [49], providing support for its role of pain modulation in depression. Similarly, another study conducted by Strigo et al. (2008) reported a negative relationship between DLPFC activity and perceived pain intensity in depressed patients subjected to painful stimulation. This finding may reflect maladaptive cortical response to pain modulation and a distinguishing factor between depressed and non-depressed individuals [46].

In summary, current evidence links the DLPFC with the affective component of pain perception in depressed patients with concomitant pain, such that a decrease in DLPFC activation is likely reflective of an impaired ability to modulate pain experience.

### Limitations and future directions

This review has some limitations that need to be addressed in future studies. Firstly, only studies reporting ALE coordinates corresponding to significant results were included in the meta-analysis. While this approach risks biasing towards significant results, our aim was to identify neural correlates that have been reported as significantly associated with pain-depression comorbidity. Although we acknowledge their relevance and contribution to the field, close examination of the non-significant ALE studies to understand the influence of various clinical and experimental factors, would be beyond the scope of this project.

Another limitation relates to the relatively small number of relevant studies available. Surprisingly, there were very few studies investigating comorbid pain and depression; when we attempted to dissociate pain with depression vs. depression with pain, we also observed a very small number of studies focusing on depression as primary diagnosis. In fact, both our ALE analyses of pain in depression and depression in pain likely are low in statistical power [23] and should be interpreted with caution. Of note is also that the majority of studies assessed for quality received the label of ‘poor’ or ‘fair’, suggesting that the level of evidence and conclusions in the review is fairly low. This could be partially due to the reporting standards for neuroimaging studies differing from clinical observational studies, for which our quality assessment tool was primarily designed for. For example, ALE analysis is limited in its ability to measure effect sizes of the effects in identified regions of difference. Related to this, our focus on the neuroimaging findings also meant that there were few longitudinal studies identified in our search. Intervention studies, such as randomised control trials of pharmacological or brain stimulation efficacy were not included due to concerns regarding methodological heterogeneity and the tendency for these studies to include region of interest imaging analyses rather than whole-brain measures. Inclusion of a wider variety of studies, such as interventional and clinical trial designs, would be important to explore in future research, to identify causal relationships between pain and depression.

Finally, although levels of depression symptoms reported in the experimental groups (i.e., participants with pain or depression) were significantly elevated compared to healthy controls, they have predominantly been either minimal or mild. This becomes relevant when findings are referenced in translational research, with its scope of application restricted to patients presenting with only subclinical depression. We attempted to explore the relationship between pain/depression severity and brain. Unfortunately, due to methodological heterogeneity we were unable to find dose-response pattern. There was not enough data for us to draw a conclusion or run specific analyses addressing this question. We only remark on the relatively mild/moderate levels of pain and depression used in previous studies.

## Conclusion

To our knowledge, this is the first ALE meta-analysis which specifically investigated neural correlates of comorbid pain and depression. In keeping with previous literature, amygdala, and its connectivity with the MPFC were associated with depressed mood in chronic pain, while hypoactivity of the left DLPFC and increased thalamic activation were linked with pain in depression, likely reflecting maladaptive attentional processing and pain modulation in depressed patients. In conclusion, our findings contribute to the understanding of the neurobiology of pain and depression by highlighting the paucity of direct comorbidity studies and the need to differentiate patients with pain conditions from those with depression in future research and clinical practice.

## Supplementary information

Supplementary Materials

## Data Availability

The full list of search terms and databases used is available in Supplementary Materials. Data extracted from the studies is presented in Table 1. The lists of MNI coordinates used for ALE analysis (by contrasts reported here) are available in the Supplementary Materials.
