# Supplementary material for: Neural correlates of co-occurring pain and depression: an activation-likelihood estimation (ALE) meta-analysis and systematic review
Source: Transl Psychiatry. 2022 May 11;12:196. doi: 10.1038/s41398-022-01949-3 (PMC9095719; doi:10.1038/s41398-022-01949-3)
Supplement: Supplementary file 1 — Supplementary Materials [file 41398_2022_1949_MOESM1_ESM.docx]

**Supplementary Materials**

List of abbreviated names of brain regions – page 2

Search terms by database – page 3

Depression scales references – page 6

Supplementary Figure 1 – page 7

Coordinates for the Primary Pain contrast – page 8

Coordinates for the Primary Depression contrast – page 11

List of abbreviated names of brain regions

(B) = bilateral

(L) = left-sided

(R) = right-sided

ACC = anterior cingulate cortex

DLPFC = dorsolateral prefrontal cortex

HPA = hypothalamus-pituitary axis

IFG = inferior frontal gyrus

MFG = middle frontal gyrus

MPFC = medial prefrontal cortex

OFC = orbitofrontal cortex

PAG = periaqueductal grey

PCC = posterior cingulate cortex

PCG = posterior cingulate gyrus

S1 = primary somatosensory cortex

S2 = secondary somatosensory cortex

SFG = superior frontal gyrus

VLPFC = ventrolateral prefrontal cortex

Search terms by database

- PsycInfo:

[exp “Depression (Emotion)*/or exp Major Depression/ or exp Reactive Depression/ or exp Beck Depression Inventory/ or exp Treatment Resistant Depression/ or depression.mp. or exp Recurrent Depression/ or exp Late Life Depression/] AND [(exp Pain Management/ or pain.mp. or exp Myofascial Pain/ or exp Pain Measurement/ or exp Pain Thresholds/ or exp Pain Perception/ or exp Neuropathic Pain/ or exp Chronic Pain/ or exp Back Pain/ or exp Pain/) OR (exp Musculoskeletal Disorders/ or exp Fibromyalgia/ or exp Rheumatoid Arthritis/ or fibromyalgia.mp.) OR (exp Trigeminal Neuralgia/ or exp Neuralgia/ or neuralgia.mp.)] AND [(neuroimaging.mp. or exp Neuroimaging/ or exp Tomography/) OR (exp Magnetoencephalography/ or exp Brain/ or exp Magnetic Resonance Imaging/ or exp Neuropathology/ or magnetic resonance imaging.mp.) OR (mri.mp. or exp Magnetic Resonance Imaging) OR (neuroanatomy.mp. or exp Neuroanatomy/) OR ((neural adj5 (pathway* or correlate* or system* or circuit* or network* or activit* or mechanism* or basis or structure*)).mp. [mp=title, abstract, heading word, table of contents, key concepts, original title, tests & measures, mesh])]

- Embase

[exp major depression/ or exp depression/ or depression.mp. or exp depression assessment/] AND [(exp chronic pain/ or pain/) OR (exp fibromyalgia/ or fibromyalgia.mp.) OR (neuralgia.mp. or exp neuralgia/)] AND [(exp neuroimaging/ or exp functional neuroimaging/ or neuroimaging.mp.) OR (mri.mp. or exp nuclear magnetic resonance imaging/) OR (neuroanatomy.mp. or exp neuroanatomy/) OR (neural adj3 (correlate* or pathway* or circuit* or structure* or network*). mp. [mp=title, abstract, heading word, drug trade name, original title, device manufacturer, drug manufacturer, device trade name, keyword, floating subheading word, candidate term word])]

- Medline

[(exp neuroimaging/ or exp functional neuroimaging/ limited to (full text and human and English language)) OR (exp nuclear magnetic resonance imaging/ limited to (full text and human and English language)) OR (neural adj3 (pathway* or system* or network* or circuit* or strucutr*) mp. [mp=title, abstract, original title, name of substance word, subject heading word, floating sub-heading word, keyword heading word, organism supplementary concept word, protocol supplementary concept word, rare disease supplementary concept word, unique identifier, synonyms] limited to (full text and human and English language)) OR (exp neuroanatomy/ limited to (full text and human and English language))] AND [((exp pain severity/ or exp pain intensity/ or exp pain threshold/ or exp chronic pain/ or pain.mp. or exp pain/) limited to (full text and human and English language)) OR ((exp fibromyalgia/ or fibromyalgia.mp) limited to full text and human and English language) OR ((neuralgia.mp. or exp neuralgia/ or exp trigeminus neuralgia) limited to full text and human and English language)] AND [((exp major depression/ or exp chronic depression/ or exp depression/) limited to full text and human and English language) OR ((depression.mp. or exp depression/ or exp major depression/) limited to full text and human and English language)]

[((exp pain severity/ or exp pain intensity/ or exp pain threshold/ or exp chronic pain/ or pain.mp. or exp pain/) limited to full text and human and English language) OR ((exp fibromyalgia/ or fibromyalgia.mp) limited to full text and human and English language) OR ((neuralgia.mp. or exp neuralgia/ or exp trigeminus neuralgia/) limited to full text and human and English language)] AND [((exp major depression/ or exp chronic depression/ or exp depression/) limited to full text and human and English language) OR ((depressive.mp. or exp depression/ or exp major depression) limited to full text and human and English language)] AND [(((exp neuroimaging/ or exp functional neuroimaging/) limited to full text and human and English language) or ((neural adj3 (pathway* or system* or network* or circuit* or strucutr*).mp. [mp=title, abstract, original title, name of substance word, subject heading word, floating sub-heading, organism supplementary concept word, protocol supplementary concept word, rare disease supplementary concept word, unique identifier, synonyms] limited to full text and human and English language) or ((exp neuroanatomy/) limited to full text and human and English language) or ((exp magnetic resonance imaging/ or mri.mp) limited to English language and full text and humans))]

[((exp Neuroimaging/ or exp Functional Neuroimaging/ or neuroimaging.mp.) limited to English language and full text and humans) OR ((brain imaging.mp. or exp Neuroimaging/) limited to English language and full text and humans) OR ((“magnetic resonance imaging” or “MRI” or “positron emission tomography” or “single photon emission computed tomography” or “SPECT”).mp. [mp=title, abstract, original title, name of substance word, subject heading word, floating sub-heading word, keyword heading word, organism supplementary concept word, protocol supplementary concept word, rare disease supplementary concept word, unique identifier, synonyms]) OR ((neural adj3 (path* or circuit* or system* or network*).mp. [mp=title, abstract, original title, name of substance word, subject heading word, floating sub-heading word, keyword heading word, organism supplementary concept word, protocol supplementary concept word, rare disease supplementary concept word, unique identifier, synonyms] limited to English language and full text and humans) OR ((neuroanatomy.mp. or exp Neuroanatomy/ or exp Neural Pathways/) limited to English language and full text and humans)] AND [((exp Chronic Pain/ or pain.mp. or exp Nociceptive Pain/ or exp Pain/ or exp Pain Perception/) limited to English language and full text and humans) OR ((exp Neuralgia/ or neuralgia.mp) limited to english language and full text and humans) OR ((fibromyalgia.mp. or exp fibromyalgia/) limited to english language and full text and humans)] AND [((depression.mp. or exp Depression/) limited to english language and full text and humans) OR ((Depressive Disorder, Major/ or depression.mp) limited to english language and full text and humans) OR ((Depressive Disorder, Major/ or MDD.mp) limited to english language and full text and humans)]

- Web of Science

[((TS=(neural NEAR/3 (path* OR circuit* OR network* OR system*))) AND LANGUAGE: (English) AND DOCUMENT TYPES: (article)) OR ((TS=((structural OR functional) AND neuroimaging)) AND LANGUAGE: (English) AND DOCUMENT TYPES: (Article)) OR ((TS=(“positron emission tomography” OR SPECT OR “single photon emission computed tomography”)) AND LANGUAGE: (English) AND DOCUMENT TYPES: (Article)) OR ((TS=(“magnetic resonance imaging” OR mri OR neuroimaging OR neuroanatomy)) AND LANGUAGE: (English) AND DOCUMENT TYPES: (Article))] AND [(TS=(pain OR fibromyalgi* OR neuralgi*)) AND LANGUAGE: (English) AND DOCUMENT TYPES: (article)] AND [(TS=depress*) AND LANGUAGE: (English) AND DOCUMENT TYPES: (Article)]

- PubMed

"depression"[MeSH Major Topic] AND ("pain"[MeSH Major Topic] OR "neuralgia"[MeSH Major Topic] OR "fibromyalgia"[MeSH Major Topic]) AND ("neuroimaging"[MeSH Major Topic] OR "brain"[MeSH Major Topic] OR "magnetic resonance imaging"[MeSH Major Topic] OR "positron emission tomography"[MeSH Major Topic] OR "single photon emission computed tomography computed tomography"[MeSH Major Topic] OR "neural pathways"[MeSH Major Topic]) AND "English"[Language] AND 1800/01/01:2020/08/15[Date - Publication]

Depression scales references

Beck, A. T., Steer, R. A., & Brown, G. (1996). Beck depression inventory–II. Psychological Assessment.

Hamilton M. A rating scale for depression. J Neurol Neurosurg Psychiatry 1960; 23:56–62

Jensen, M. P., Chen, C., & Brugger, A. M. (2003). Interpretation of visual analog scale ratings and change scores: a reanalysis of two clinical trials of postoperative pain. The Journal of Pain, 4(7), 407-414.

Kroenke, K., Spitzer, R. L., & Williams, J. B. (2001). The PHQ-9: validity of a brief depression severity measure. Journal of general internal medicine, 16(9), 606–613. https://doi.org/10.1046/j.1525-1497.2001.016009606.x

McCaffery, M., & Beebe, A. (1989). The numeric pain rating scale instructions. In Pain: Clinic Manual for Nursing Practice. Mosby, St. Louis.

Snaith, R. P. (2003). The hospital anxiety and depression scale. Health and quality of life outcomes, 1(1), 1-4.

Yang, M., Rendas-Baum, R., Varon, S. F., & Kosinski, M. (2011). Validation of the Headache Impact Test (HIT-6™) across episodic and chronic migraine. Cephalalgia : an international journal of headache, 31(3), 357–367. https://doi.org/10.1177/0333102410379890

Zung, WW (1965) A self-rating depression scale. Arch Gen Psychiatry 12, 63-70.

Supplementary Figure 1. PRISMA Flowchart (August 2020-September 2021 search)


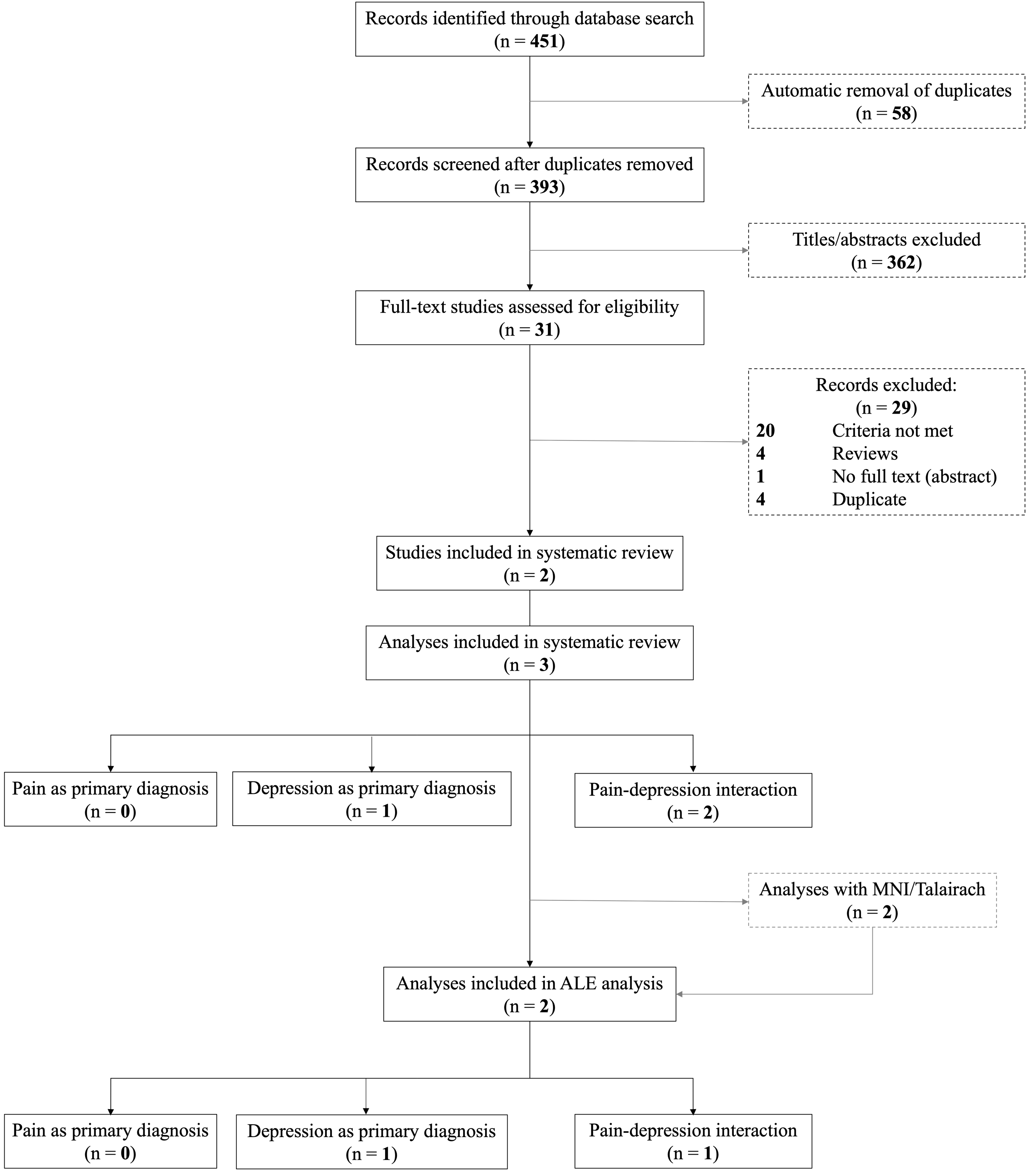


Coordinates for the Primary Pain contrast

// Reference=MNI

// Albrecht 2019

// Subjects=25

2 40 -2

-10 36 22

24 22 50

// As-Sanie 2016

// Subjects=14

34 19 0

0 46 22

// Cifre 2012

// Subjects=20

20 -18 12

6 -32 -10

-2 8 30

-6 -32 -10

// Feliu-Soler 2020

// Subjects=47

11 -2 -12

-8 0 -11

// Giesecke 2005

// Subjects=30

-20.39 0.01 -17.22

20.23 0.43 -15.49

35.38 4.69 10.75

// Gustin 2013

// Subjects=42

-14 -18 -4

6 -6 0

6 38 2

-42 22 22

10 -36 30

-26 -42 0

26 -40 -6

-12 -4 8

6 -4 0

-48 24 38

0 -42 28

-28 28 12

-38 -36 -10

36 -42 -2

// Ikeda 2018

// Subjects=23

32 10 -18

-12 12 -12

// Jensen 2013

// Subjects=26

18 -72 5

38 -62 -18

34 -46 -14

24 -86 17

17 -26 39

-39 -70 44

-25 -2 45

-18 -77 30

-37 -29 -25

-54 -51 27

21 15 0

// Khan 2014

// Subjects=18

-9 57 3

35 3 -24

20 -2 -21

// Klega 2010

// Subjects=10

-52 -22 2

// Li J 2020

// Subjects=49

-33 42 22.15

-30 28.5 -9

30 51 -7.5

-25.5 57 -6

37.5 60 4.5

-33 6 15

// Li J 2020

// Subjects=64

-28.5 28.5 -12

// Ma 2018

// Subjects=32

12 -12 6

27 -3 -42

// Rosenberger 2013

// Subjects=15

20 -40 -28

14 -34 -14

8 -62 -16

26 -56 -34

38 -48 -40

-2 -82 -44

46 -52 -56

36 -60 -62

// Schweinhardt 2008

// Subjects=20

-4 66 8

// Seifert 2011

// Subjects=13

-35.25 16.35 2.77

// Vachon-Presseau 2016

// Subjects=39

-23 -5 -18

23 -5 -18

-10 12 -7

10 12 -7

-7 66 4

8 66 5

// Wagner 2009

// Subjects=32

24.66 -16.58 -1.85

-24.52 -20.48 -6.53

// Wang 2017

// Subjects=38

39 -1 15

-6 50 9

// Zhang 2018

// Subjects=29

18 4.5 -22

36 54 0

Coordinates for the Primary Depression contrast

// Reference=MNI

// Bar 2007

// Subjects=26

-15.87 -9.45 11.4

// Berna 2010

// Subjects=40

-30 -58 48

-6 -46 44

36 -40 38

-62 -44 30

-56 14 6

-20 -64 64

-24 22 44

-2 6 -6

-45 40 -14

-56 -56 -10

-56 -62 -26

-36 -26 8

-20 -36 -2

-16 -12 8

-12 10 6

-32 -76 28

-38 -32 34

-68 -36 -4

-66 -42 -6

52 -12 36

2 -48 44

-56 -18 32

// Malejko 2020

// Subjects=22

40 -24 54

// Strigo 2008

// Subjects=30

30.77 -2.11 -35.47

-41.76 -11.03 -20.51

-53.39 -53.74 -21.25

5.22 41.55 -5.38

12.79 45.79 7.56

36.69 17.04 46.5

-45.6 12.14 45.62

-49.24 38.3 -1.14

-17.83 31.86 50.34

-22.12 24 45.47

-56.47 -43.67 -.87

-34.65 -69.73 45.67

-48.66 -50.06 39.72

11.8 -35.75 -14.59

13.85 -40.99 -27.55

Additional search studies:

//Hou 2021: activation during pain stimulation DEP vs. HC

// Subjects=44

-64 -6 12

39 0 -45

52 -8 -4

//Hou 2021: activation during pain stimulation DEP+P vs. DEP

// Subjects=36

-56 -22 42

-39 -63 -15

-52 -12 -10
